# Migration and sustainable development

**DOI:** 10.1073/pnas.2206193121

**Published:** 2024-01-08

**Authors:** William Neil Adger, Sonja Fransen, Ricardo Safra de Campos, William C. Clark

**Affiliations:** ^a^Department of Geography, Faculty of Environment, Science and Economy, University of Exeter, Exeter EX4 4RJ, United Kingdom; ^b^United Nations University–Maastricht Economic and Social Research Institute on Innovation and Technology, and School of Economics and Business, Maastricht University, Maastricht 6211 AX, The Netherlands; ^c^Global Systems Institute, Faculty of Environment, Science and Economy, University of Exeter, Exeter EX4 4RJ, United Kingdom; ^d^Sustainability Science Program, Harvard Kennedy School of Government, Harvard University, Cambridge, MA 02138

**Keywords:** sustainable development, migration, demographic change, natural resources, mobility

## Abstract

To understand the implications of migration for sustainable development requires a comprehensive consideration of a range of population movements and their feedback across space and time. This Perspective reviews emerging science at the interface of migration studies, demography, and sustainability, focusing on consequences of migration flows for nature-society interactions including on societal outcomes such as inequality; environmental causes and consequences of involuntary displacement; and processes of cultural convergence in sustainability practices in dynamic new populations. We advance a framework that demonstrates how migration outcomes result in identifiable consequences on resources, environmental burdens and well-being, and on innovation, adaptation, and challenges for sustainability governance. We elaborate the research frontiers of migration for sustainability science, explicitly integrating the full spectrum of regular migration decisions dominated by economic motives through to involuntary displacement due to social or environmental stresses. Migration can potentially contribute to sustainability transitions when it enhances well-being while not exacerbating structural inequalities or compound uneven burdens on environmental resources.

## Sustainable Development and Migration Interactions

In the contemporary globalized era, the prospects for sustainable development are affected by the movement of people across the world, but this phenomenon is not central to many accounts of sustainability science. The normative goal of sustainable development is well understood as “the enhancement of well-being in ways that more equitably meet needs of present and future generations.” The equity dimension is most commonly conceived of as incorporating the capacity and capability to enact desired futures. Sustainability science seeks to promote that goal through better understanding of how nature–society interactions create complex adaptive systems operating across a wide range of temporal and spatial scales. These systems mediate efforts to tap the world’s environmental and social resources to generate human well-being (1). A central insight of that research program that we draw on here is that conservation of the earth’s life support systems can most usefully be seen as a necessary, though not sufficient, means for achieving the ultimate ends of sustainable development, i.e., of equitable improvements in human well-being. One focus of sustainability research has been on how connections among places, as one set of complex connections between society and nature, shape development pathways through processes such as trade, investment, the spread of ideas, biological invasions, and pollution flows (2). Connections between places are clearly also made by flows of people between them. Contemporary development pathways more than ever involve mobile human populations moving to avail themselves of economic and life opportunities, as well as to escape social conflict and environmental stress, yet such connections remain unaddressed in much-coupled systems research.

This historical neglect, however, is beginning to change. A growing body of research is exploring the significance of movements of people for sustainable development. Environmental change is increasingly understood to have been central to the movements and well-being of people everywhere and always: examples range from opening up opportunities for advancing settlement frontiers as conditions become more favorable or where new resources are discovered (3), through to cases of population collapses when resources are over-exploited, trade changes or conflict arise (4). Likewise, the movement of people has been shown to have myriad impacts on natural resources and environments.

To date, however, this research has been disproportionately focused on movements involving flight from disaster. It has been less focused on a comprehensive assessment that also includes the dominant role of people’s movement in seeking positive opportunities to enhance their well-being. And it has had more to say about impacts on places and peoples that receive migrants than about the places and peoples that migration leaves behind. The goal of this Perspective is to provide a balanced synthesis of how human migration is coming to be understood to shape the prospects for sustainable development. We seek to open up the sustainability sciences to these opportunities for new data, integration, and enhanced explanations of nature–society coevolution.

### Global Migration Trends.

Migration as used here is the movement of people measured in terms of their primary place of residence, that includes substantial internal movement within countries and international movements between countries. In this paper, we refer to migration as people moving their permanent place of residence for a significant distance, across jurisdictional boundaries (even within countries), for more than 1 y and migrants as stocks resulting from those flows of people. Migration is part of a mobility continuum that includes temporary moves ranging from daily commutes through to permanent relocations.

**Fig. 1.**
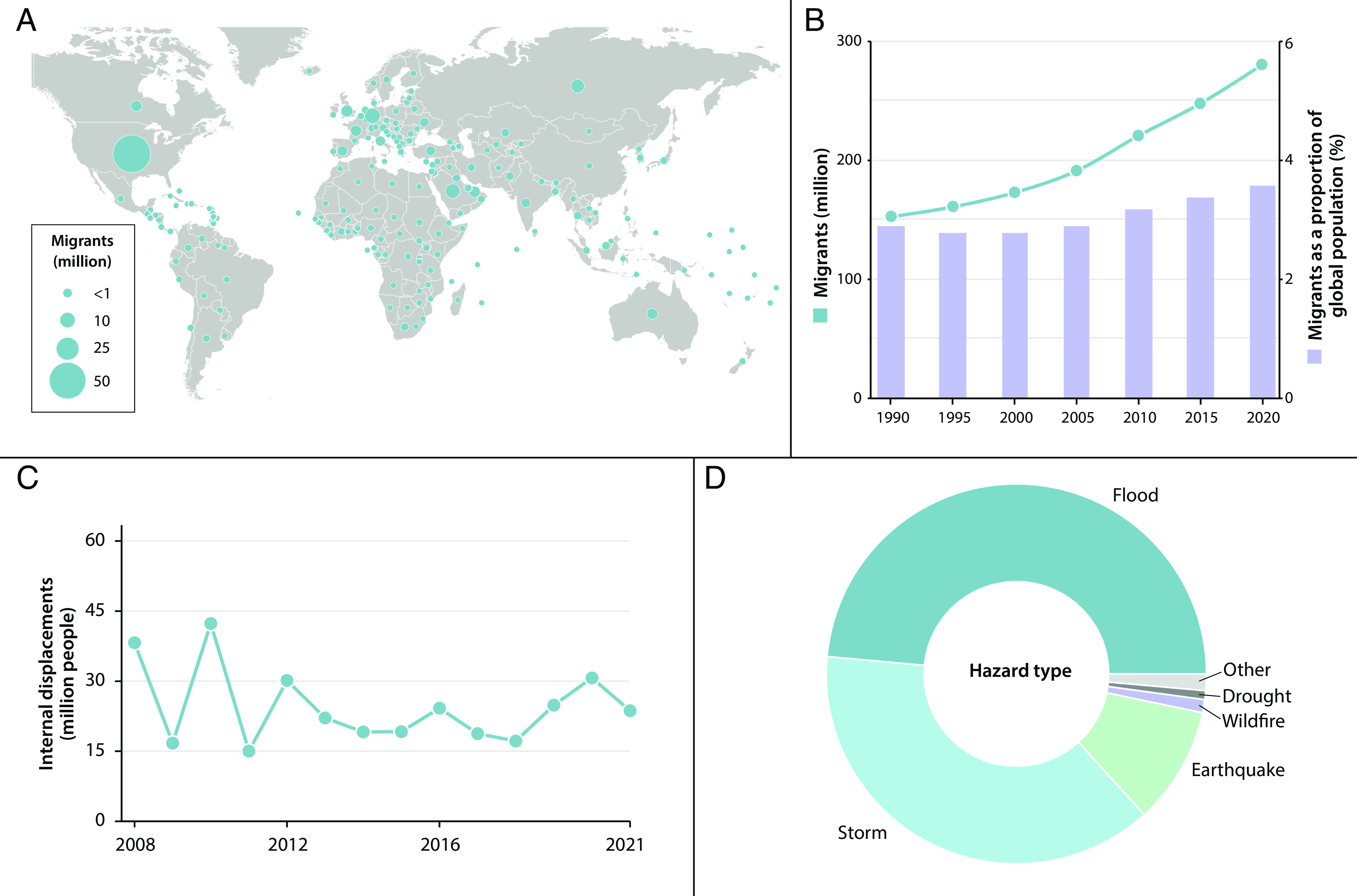
Trends in stocks of migrants globally with trends in displacement from natural hazards: (*A*) Immigrants (million) as at 2020 (7); (*B*) International migrants (million) (left axis) and as proportion of global population (right axis) (1990 to 2020); (*C*) Aggregate flow of people displaced internally within their own countries in each year (2008 to 2021) (8) (*D*) Estimated causes of internal displacements involving natural hazards in 2021 (excludes involuntary refugee migration from conflict) (8).

A global picture of the magnitudes of migration in the modern world remains less sharp than would be desirable due to a host of methodological and data comparability issues. Recent studies, however, have provided good indications of international and internal migrant stocks (5, 6). The data show that, in absolute terms, there is now a larger stock of lifetime migrants, individuals who are residing in a place other than where they were born than at any point in human history. These stocks are dominated still in the opening part of the 21st century by within-country movements of people into urban settlements, driving the global urbanization trend. While quantitative estimates of the number of internal migrants within larger countries vary, depending on definitions, they are almost certainly reaching 800 million people, about 10% of the world’s population (5). International migrants are a smaller fraction—about one in thirty of the global population—and mainly clustered to movement to large open trading economies (Fig. 1*A*) (7). Numbers of voluntary international migrants are growing in absolute terms, rising to approximately 280 million in 2020, with the percentage of the total population also rising steadily from 2.9% in 1990 to 3.6% in 2020 (Fig. 1*B*) (7). The number of migrants is likely to continue to grow despite a temporary reduction caused by the COVID-19 pandemic (6).

There is significant spatial disparity in the distribution of migrant populations across the planet, partially explaining their neglect in goal setting for sustainable development. In some places, migrants are so infrequent as to have little to no impact on nature or society, while in others, they are so substantial that they dominate sustainability dynamics. Consider, for example, that most of the voluntary within-country movement of people is away from a very large number of small rural settlements toward a relatively few large urban ones, with very different consequences than if migrants had been spread evenly across the land. Migration of people displaced by social or environmental stresses tends to take place over relatively short distances. Given the concentration of such stresses in a relatively few parts of the globe, refugees, and other involuntary migrants tend to be similarly clustered. At the end of 2022, 52% of global refugees came from just three countries (the Syrian Arab Republic, Ukraine, and Afghanistan), and 70% of all refugees worldwide were hosted in neighboring countries (9). And for international migrants seeking a better life, as noted above, movement is largely directed toward large open trading economies. European and Asian countries with high labor demands are the largest destination for voluntary international migrants combining for 61.4% of people residing outside their country of birth (followed by North American countries with 21%); while Oceania has the largest share of international migrants as a proportion of the total population (6). These trends are hugely significant for sustainability, beyond the subset of people who have moved involuntarily. In summary, it is less global averages of changing migration flows and migrant stocks and more their extreme spatial heterogeneity that makes them so important for the pursuit of sustainability.

Here, we seek to address migration processes and the main trends and drivers on a broad spectrum from regular migration to involuntary displacement and immobility. Regular voluntary movement is common in all societies, as highlighted in Fig. 1. It is characterized by diversity and is part of broader demographic, geopolitical, technological, economic, and environmental transformations. The World Bank’s 2023 World Development Report argues that migration is currently vital to economic progress for countries at all income levels due to demographic changes such as aging in some regions and population growth in others (6). The Report argues that international cooperation is key to realizing the benefits and avoiding the harms of social conflict and brain drain between regions. Migration, and linkages between source and destination areas, represents one of the most significant economic phenomena: International remittances and return migration are frequently cited as dwarfing official government transfers or even trade flows between global regions (10).

In parallel, public policy discussions on migration most frequently focus on involuntary displacement. The main policy focus is the subset of refugees—defined in international treaties as people moving involuntarily as a result of conflict and persecution: Refugee stock numbers have fluctuated between 0.1 and 0.3% of global population in the past half century, fluctuating principally through levels of conflict and political oppression in source countries (11). Conflicts in Sudan and Syria have been held up, despite contested evidence, as being aggravated by environmental scarcity and climate change (12). Yet the wider evidence shows that the principal drivers of refugee movements remain political violence and repression, with environmental scarcity or climate change being a lesser mediating factor (13, 14). That said, climate and other environmental changes are increasingly affecting, in diverse ways, involuntary movement. Involuntary movement from weather disasters, for example, is increasing decade-on-decade. On average, depending on the method for estimation, more than 25 million people were displaced internally each year in the past decade (Fig. 1*C*) (8, 15). Fig. 1*D* demonstrates that most involuntary displacements resulting from environmental stresses are due to floods and storms (8). Over 30 million people were directly affected by floods in Pakistan in 2022, for example, with many millions of those becoming displaced for a number of weeks or even months, with the vast majority returning home.

There are real risks of increasing further flows in involuntary displacement leading to more permanent migration as a result of environmental degradation. Projections of numbers of people involuntarily displaced, even on a temporary basis, consistently show increases due to escalating trends in weather-related extremes—from floods, drought, and wildfire. In addition, slow-onset climate impacts (e.g. water stress and sea level rise) are growing and could increase the number of internal migrants by more than 100 million by 2050 (16).

But the policy focus most often assumes large numbers of people moving en masse across large distances and even across national borders. The reported numbers of prospective environmental migrants assume that all populations facing environmental risks will move (17) and portray migration as principally an issue of border security (18). Estimates of future habitability, for example, map where current populations will face future risks: Xu et al. (19) estimate that up to three billion people currently reside in areas of the world which are likely to experience climates during the 21st century outside the historic envelope of habitable places. Yet this does not translate into populations on the move—rather to downward trends in the habitability and likely investment attractiveness of cities, leading to long-term changes in population densities (20). Many studies demonstrate that populations remain in place despite significant environmental risks because of individuals’ attachment to place (21). Jarillo and Barnett, for example, show that despite growing environmental challenges, a sense of belonging and responsibility acts as a centripetal force to incentivize continuity and maintains populations in place. In essence, rather than a wave of climate migrants, a more appropriate analogy is the slow-changing tide of redistribution of settlements and population (22).

### Drivers of Migration and Migration Decision-Making.

At the fundamental level, across both regular and involuntary migration, individual decisions to move are facilitated or constrained by economic, social, technological, and environmental factors. This view is at the core of every model and description of migration decision-making, from behavioral models to economic household models (23). The aggregation of these myriad individual decisions and successive movements of people (or decisions to stay) affect the pathways of development for those individuals, as well as their origin and destination communities and societies.

Migration studies in the past decade have pointed to the on average positive benefits of migration across the lifecourse for those involved, measured in multiple dimensions such as perceived well-being (24). Migration and urbanization processes are intensifying globally, and particularly in low-income and middle-income countries, not primarily because of political or environmental stress, but rather because movement toward economic opportunities increases life chances and potential material well-being.

Models of stages of development and demographic change suggest that the capacity to move is enhanced through increased financial, social, and human capital. Increasing levels of migration observed in most world regions are perceived to be a direct result of economic opportunities, not least through industrialization. At higher levels of per capita income, incentives for emigration begin to decline (23), with contemporary global flows remaining dominated by internal movement to cities, and well-established migration corridors for skilled migrants. Migration is perceived to be constrained by insufficient resources, and the effects of climate change and other environmental degradations on poverty and growth may already have affected the ability of people to move, constraining their life chances (25).

### Implications for Sustainability.

Population movements have diverse implications for sustainability, both through impacts on the underlying social or environmental resources that constitute the foundations for long-term development and more directly through immediate changes in people’s well-being. The outcome of environmental dimensions and immediate economic benefits demonstrate potentially counterbalancing elements. First, the movement of labor is central to economic globalization trends. Labor movement leaves those economies experiencing net out-migration with lower levels of working-age adults, and potentially with skills shortages and reduced public revenue, while at the same time, remittances of finance from overseas migrants provide funds for investments. There is some evidence that the aggregation of all migration economic affects (meeting skills gaps, remittance flows, brain drain, and return migration) are net positive and many times the benefits of international trade deregulation (26, 27).

Second, migration may enhance the security of individual life course trajectories, through involuntary movement away from social or environmental risks. The evidence to date suggests that migration is an effective and common response to environmental degradation: effective in the sense of enhancing individual well-being on average, yet often amplifying existing inequalities in terms of access to resources and options for migration (18).

Third, changing population densities in specific areas, impacted by international movements, urban growth, and industrialization, can have a variety of impacts on the underlying environmental resource base (28). Migration flows to cities have been shown to reinforce spatial inequality, through clustering of recent migrants in particular in socially disadvantaged neighborhoods. It is these processes of spatial inequality that make the achievement of many sustainability goals in cities so challenging (29). Yet there is also evidence of negative correlations between clustering of populations, reinforced by migration, and environmental burdens such as air pollution which are often less on a per capita basis in urban than in rural areas. In addition, migrants are attracted to cleaner and greener destinations and often to be part of transformative sustainability changes: Squali (30) shows for example such a relationship for US states over the past decades. Other studies suggest that immigrant populations exhibit pro-social and pro-environmental behaviors as part of identity formation and for integration into host societies (31). Head et al. (26) document how immigrant populations build new hybrid identities through bringing food cultures and agricultural practices into urban Australia. At the same time, the impacts of emigration affect the prospects for sustainability. Emigration can reduce pressures on the environment and resource base of places where populations are declining. But it can also leave those places with too few of the kinds of people needed to make productive and sustainable use of local resources, resulting in downward trajectories of well-being for populations with higher proportions of women, children, and elderly that migration tends to leave behind (27).

Hence the consequences of migration result from the interaction of these trends: in individual life outcomes of migrants, of macro-economic consequences of aggregate flows, of challenges of social integration, and of transformations of the underlying resource base. The overall implications of those consequences for sustainability depend on how well migrants are integrated into economic and civic life in destination regions, how remittance flows are regulated, and how changing population densities affect the environments of both source and destination places.

## Relationships between Migration and Pathways of Sustainable Development

The overall significance of migration for sustainability involves environmental and social outcomes and changes in long-run prospects for people moving to places where they can increase their economic and life opportunities, or from places where their livelihood security is threatened by social or environmental stresses. At the individual level, migration decisions are adaptation responses to potentially disruptive change and differential opportunities. Aggregate migration flows represent potential long-term transformations in economies: shifts from one regime and its associated development pathways to another, that include significant environmental footprints of growing or diminishing populations (30). The potential to transform economies is more limited where people choose to remain in place, or there are high levels of involuntary immobility. And factors determining who is mobile and who is not can significantly influence whether migration supports or undermines the central equity components of sustainability goals.

### A Framework for Assessment.

Understanding the relationship between migration and sustainability involves an aggregation of impacts on society, economies, and environmental resources across space and time. A comprehensive and holistic assessment of these interactions necessarily includes consideration of places of origin, places of destination, and the decisions for voluntary and involuntary movement between them. It requires systematic accounting for the impact of migration on the environment and on well-being not only for the individuals migrating, but also at the collective societal level and across the long run. The long-run focus of sustainability goals in turn implies that a useful accounting must capture consequences of migration for the resource base on which development ultimately depends.

**Fig. 2.**
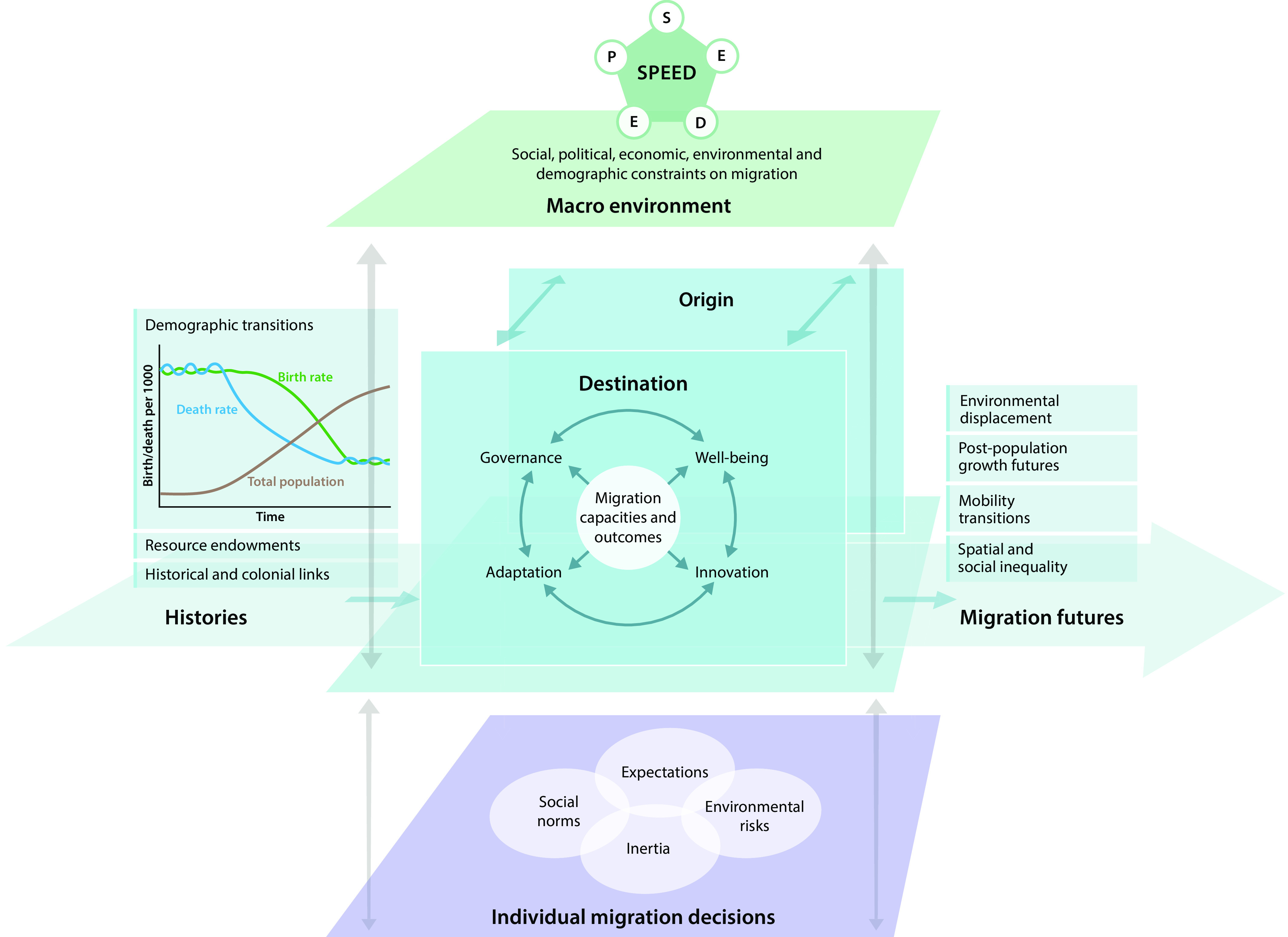
How migration affects the NSS and their future trajectories: The interplay of macrolevel structures, place-based processes, and individual level decisions (modified from ref. 1).

Multiple relationships between migration and sustainability are illustrated in Fig. 2. The overall consequences of migration for sustainability depend on the potential for migration to affect desirable change in nature–society interactions in both origin and destination nature–society systems (NSSs) (*Central*). Migration outcomes in places of origin and destination depend on the initial well-being of migrant and non-migrant populations and their respective resource endowments, on their capacity for innovation, on their capacity to adapt, and on their capacity for governance. For every locality and for every migration flow, local possibilities are constrained by overarching structures (*Top*): political structures such as border control and discourses; demographic structures (population aging and demand for labor); environmental conditions, and economic benefits. The direction and clustering of aggregate migration flows are primarily determined by historical factors (*Left*) and made up of millions of individual migration decisions (*Bottom*) constrained by the prevailing social norms and practices of migration. These elements are all informed and core to research in sustainability science and recognized in scholarship about how migration impacts nature–society dynamics and the well-being they support (32).

What then is the evidence for how contemporary and historic migrations flows interact with these factors? Well-being, innovation, adaptation, and governance are core elements of the potential to incorporate migration in studies of nature–society interactions and so better to illuminate the prospects for sustainable development.

### Migration and Well-Being.

In the context of sustainable development, well-being is of interest both as a determinant and as an outcome of migration flows. Turning first to the role of well-being as a determinant of migration, recent theoretical advances link migration and well-being through a capabilities and aspirations framework (33). Such approach distinguishes the migration process into two intrinsically connected phases: the aspiration to migrate and the capability to migrate (the effective opportunities individuals have to fulfill their migration aspirations).

Aspirations are the emotional constructs that represent what the future might or should look like derived from previous migration experiences from the migrants’ personal knowledge and their own social network (34). Migration involves the capability and freedom to choose where to live or to stay put. This concept builds on Sen’s (35) concepts of resources and public goods as giving freedom to choose to stay or move beyond static push models. Capabilities for migration include sufficient resources and finances, alongside networks and social capital that make it easier for people to search for economic opportunities and residence in destination regions.

There is strong evidence that migration can increase net well-being for those involved, even accounting for net migration across origin and destination places. The evidence on migration leading to a more satisfying life ranges from economic models of labor market efficiencies through to empirical studies on the implication of migration on subjective well-being, and material well-being (24). It is also well established that migration can foster well-being in the form of upward social mobility, the relative improvement in material living conditions and in social status, as experienced by individuals and families (36).

Migration flows do not, however, necessarily promote equitable increases in well-being, and thus sustainable development. Migration to places of more intense economic activity can certainly accelerate overall environmental degradation and thus undermine intergenerational equity. And at least in the short run, migration flows create challenges for social cohesion, exacerbate spatial and social inequality and exclude political voice for migrants resulting in constrained shared visions of civic life (37). Migrants, especially low-skilled migrant populations, are exposed to insecurity and social exclusion in many places of destination in every part of the world and may be subject to significant constraints on upward social mobility. Studies have shown high levels of stress and anxiety in migrant populations in Chinese cities, for example, because of perceived marginalization and exclusion (38). This is referred to as the “miserable migrant effect”: that even with increased economic situation and prospects, the insecurity of city life has detrimental effects on perceived well-being, perceived autonomy, and, in turn on under-reported mental ill-health (39).

In summary, the available evidence suggests that the overall effect of migration on well-being and sustainable development pathways remains ambiguous, depending on the time and spatial scale examined. As is so often the case in the pursuit of sustainability, outcomes are shaped by global trends but depend on local context, including attributes of particular origin and destination places, and of particular potential migrants and their social networks.

### Processes of Linkage: Innovation and Adaptation.

Migration has significant potential for innovation, adaptation, and transformation through social remittances and changing demographic compositions. Social integration of new migrant populations into destination societies, and its effects on social cohesion, is a significant sustainability issue. The process of social integration is linked to issues of identity, language, and culture. As a consequence of immigration, destination societies are becoming ethnically more diverse, a process which is intrinsically linked to transformation toward sustainability. In the short run, migration and associated increases in ethnic diversity typically lead to lower levels of social trust and social cohesion (40, 41). Ethnic diversity has, in some circumstances, shown to have consequences for economic growth and risks of violent conflict (42). Moreover, during economic downturn, migrants are often a lightning rod for social concerns about housing availability, demand for public services and employment opportunities, even in the absence of causal relationships between those issues and migrant arrivals or presence (43). In the long run, however, ethnic diversity might have positive economic and cultural effects on destination societies (43). As pathways toward sustainability of communities and places, shaped for example by sustainable practices, are intrinsically linked to social cohesion, place attachment, and community awareness (44), the social inclusion of newcomers is an important condition for migration to contribute to innovation and adaptation.

Migration can be a demographic life-line in many countries with aging populations: International flows have become major drivers of population change (45). A stark example of such trend suggests that for the period between 2000 and 2020, the contribution of international migration to population growth in high-income countries far exceeded the balance of births over deaths (46). This has implications for dependency ratios in countries where population aging and shrinking labor forces (47). By contrast, Sub-Saharan Africa will account for the majority of the growth of the global population over the coming decades due to much higher fertility rates (46). In countries where the youth bulge has the potential to become a demographic dividend (48), the ever-present challenge of brain drain dominating flows of high skilled individuals to industrialised countries could hinder or limited development of poorer part of the world if policies are not tailored to address current migration imbalances (49).

Remittance flows are a major link between source and destination areas, and represent significant investment flows to many growing economies (50). The volume and salience of financial remittances is well established: The total amount of migrant remittances is more than three times the amount of overseas development assistance globally (51). In some cases, financial remittances have been shown to stabilize livelihoods, allowing communities to rise from poverty through investment in human and productive capital, and promote economic growth (52). There are, however, consequences for the incidence of income poverty for migrant households that do not receive remittances. In these cases, the loss of labor supply and domestic earnings of migrants are not financially compensated which potentially stagnates or deteriorates households’ living standards (53).

Remittances also take the form of flows of new ideas, human capital, and practices. There is growing a recognition that cultural shifts through such so-called social remittances have benefits that offset negative consequences of brain drain of skilled working-age adults. Positive externalities, in the form of ideas, and socio-political norms often associated with return migrants and transnational families, have been shown to foster innovation, dynamism, and entrepreneurship (54). Sakdapolrak et al. (55) show that deep linkages between origin and destination regions are indeed manifest as trans-local livelihoods—people with one foot in many places—with major potential benefits for the resilience and innovation in all of them.

In addition to positive innovation, migration has increasingly been shown to be an effective adaptation for individuals and societies facing external shocks including climate-related risks. Emigration reduces pressure on local resources, inducing financial and social remittances from migrants to their communities of origin, thereby enabling remnant populations to persist in the face of environmental hardship (56). Migration represents a demographic adaptation that can overcome critical challenges of aging populations in many destination regions. Most major migration flows are, for example, dominated by working-age adults who are on average healthier than recipient populations. This results in lowered increased dependency ratios for migrant destination regions and increased burdens of dependents in net migration outflow areas resulting from aging populations (57).

Does building adaptive capacity lead to reduced migration? Conceptual and empirical work suggest that adaptation measures have indeed reduced migration pressures in communities of origin (58). Investments of remittances in education, other forms of human capital, and agricultural adaptations, have secured and stabilized depopulation in rural Thailand, for example (53). A key challenge in preparing for climate-related migration and displacement is to identify, anticipate, and react to such flows before their occurrence (59). A further long-term challenge is identifying how adaptation through migration potentially shifts risks across time and space pushing the hazards onto other people in other places or generations. Such shifts have consequences for future risks in destination regions as a direct consequence of heighted exposure (60) to environmental risks (61) and for immobile individuals and communities (62).

Does out-migration make sustainability more likely to be realized in places depopulating due to migration? While studies suggest that increased population has undeniably contributed to environmental degradation and land use change in parts of West Africa and Asia through intensification and conversion of agricultural land (63, 64), there is less certain evidence on the impact of decreasing populations. Remittances are suggested as one of the mechanisms through rural out-migration can accelerate sustainability transitions (65), though the evidence is mixed. Concepts such as teleconnections (66) and trans-local livelihoods (55), for example, offer process-based frameworks to examine how flows of financial capital, people, materials, ideas, energy, and waste connects multiple urban and rural systems, with direct consequences for left-behind populations. The consequences of out-migration include reduced demand on resources and potentially pollution loading, but with significant demographic shifts, brain drain, and reduced labor availability.

### Governance of Migration.

Governance—how people can work together to achieve what they cannot achieve on their own—has long been a central focus of both migration and sustainability studies. And it is well established that both the governance of sustainability and the governance of migration affect migration processes and lead to altered development pathways, sometimes in unforeseen directions. For example, restrictions on international movement directly affect the availability of both skilled and unskilled labor in source and destination areas. And most international policies affecting movement of people concentrate on ensuring safe and orderly mobility and the integration of migrants in destination societies, striving for equitable improvements in well-being often in the face of protectionism and xenophobia (67).

That said, the transformative character of migration for development is not fully reflected in several of the targets and indicators that are most relevant for sustainability processes, including poverty alleviation, inequality, and environmental conservation. In particular, migration has been neglected in efforts to foster better governance of sustainability. Zickgraf et al. (68), for example, based on empirical research across North America and Europe, report that migration is often not perceived to affect sustainability outcomes by policy makers and opinion formers. More broadly, detailed analyses show that minimal attention has been paid to migration within the formalized structures of the UN’s Sustainable Development Goals (SDGs), and before them the Millennium Development Goals (69). Sustainable development is portrayed in the SDGs as a sedentary, place-bound process that is threatened by increasing mobility patterns, and particularly the movement of lower-skilled migrants (70). By not accounting for migration’s potential contributions to environmental and social outcomes, consequences for enhancing equity and for stimulating innovation make the achievement of many SDGs seem less attainable that a fuller migration-inclusive assessment would suggest (71).

The essential broader assessment of migration governance for sustainability would need to deal with a number of tensions. For example, migration creates opportunities for meeting targets for poverty reduction (SDG1) but creates challenges through new inequalities. Many policies aimed at poverty reduction do not specifically acknowledge the important role that migration pathways play in sustaining economies and populations in origin and destination areas. Yet, forms of temporary and permanent migration have been shown to be key in specifically improving the lives of poorer populations worldwide. Financial remittances, for example, being “the most tangible and least controversial link between migration and development” (70, p.1) (72) are a relatively stable source of income for many households and communities in developing countries and are generally associated with poverty reduction and increasing development in various world regions (52). To account for this development potential of migration, the SDG targets of “Ensuring equal rights to economic resources and access to basic services” (SDG target 1.4), and “building resilience of the poor” (SDG target 1.5), should therefore incorporate indicators such as equal access to mobility options to fully account for the reciprocal relationship between mobility and well-being.

Similarly, the governance of migration poses critical challenges to the SDG of Inclusive, safe, resilient, and sustainable cities (SDG11) as the principal destination of migration flows; while implications for places of origin have not yet been examined in an integrated manner taking in to account, for example, the hollowing out of certain demographic groups (73). Population growth through inward migration creates opportunities for innovation and increased well-being, but highlights resource, pollution, and housing issues for growing cities (74). Yet, the reality of migration being an important driver of urban expansion is not fully acknowledged in SDG11 targets (75). Migrants are indirectly included in the targets that stress inclusive development to “leave no-one behind” (76): “Those whose needs are reflected in the Agenda include all children, youth, persons with disabilities […], people living with HIV/AIDS, older persons, indigenous peoples, refugees and internally displaced persons and migrants” (77). However, the question that remains is how, and to what extent, the processes of increased well-being, adaptation, and innovation can be incorporated into governance arrangements when integrating new populations into urban settlements, so that sustainable, safe, and resilient localities are indeed inclusive.

Incorporating migration considerations into the governance of climate action (SDG13) also poses unmet challenges due to the irregular character of migration and displacement. Evidence on the relationship between climate change and migration and displacement is contested (14, 78). Yet it is clear that governance of the climate crisis will necessarily require focus on the conditions through which irregular migration and displacement can lead to adaptation and sustainable development in origin and destination areas. Focusing on the factors driving displacement, Vestby et al. (79) show how displacement levels are higher in more economically and politically vulnerable contexts, and thereby point to the important link between human development and the human costs of weather-related disasters.

Building better governance arrangements for sustainability will be a long-term process. But it could include many reforms in existing arrangements to better address migration. These would certainly include new protocols on climate migrants under the UN Framework Convention on Climate Change. They also could be cast amendments to the UN Global Compact on Safe and Orderly Migration (80). And a variety of regional agreements could focus on how sustainability is affected by visa and rights for movement between adjacent countries, as is increasingly being done in the Pacific region (81).

## A Forward-Looking Science for Migration and Sustainability

Migration interacts with every element of sustainable development through its role in creating well-being and meaningful lives and its impacts on natural resources, through processes such as adaptation and innovation, and through many deliberate and unforeseen consequences of both migration and sustainability governance. The outcomes of migration flows may have the potential to create substantial synergies as well as risks for societies and economies to move toward sustainability, and these need to be incorporated into scientific models. In particular, the everyday nature of migration, its role in shaping long-term development trajectories, and its relations to structural inequalities are potentially important elements and relationships in all sustainability studies (82).

The principal types of migration lead to key challenges for sustainability science and policy:

*Regular migration dominated by economic motivations:* Challenges for sustainability include resource demand shifts, with greater pressures on resources—especially but not exclusively environmental ones—in destination regions, amplified by increased resource flows to higher consumption regions. Social challenges include demographic shifts such as hollowed-out populations in origin areas, brain drain, and the potential for social conflict and increased inequality in destination regions. For example, in some cultural contexts, left-behind women take on additional economic responsibilities in addition to their roles of mothers, partners, and caregivers for other family members (83, 84).

Policy priorities in this area are to show how the pursuit of sustainability could be advanced by efforts to regularize migration, ease restrictions on return movement, and integrate populations into planning for sustainability, especially where there is a strong match between interests of origin and destination regions (6).

*Planned relocation and managed retreat made necessary by degradation of earth’s life support systems:* Challenges to sustainability of such environmentally-forced movement include disruption and loss of habitats, income, social networks, and cultural heritage. Policy priorities for helping resettlement may contribute to sustainability by focusing on accountable governance and inclusive processes that engage all affected communities in planning for their own future. This way, relocation initiatives promote equality, fairness, and resilience in affected communities (85).

*Involuntary displacement and refugees resulting from human conflict:* Such movements represent breaches of fundamental rights and from a sustainability perspective involve loss of well-being from involuntary and disrupted economic development. There are increased resource pressures and social disruption on refugee-hosting regions from long-term refugee camps or dispersal in cities and settlements. Key policy priorities are to minimize such flows through conflict avoidance, co-operation between neighboring countries on rights to move, humanitarian assistance to avoid permanent displacements, and the provision of human rights, such as the right to work and freedom of mobility, to displaced populations (86).

A challenge for sustainability science is to reset common narratives of migration from a threat to society, toward an evidence-oriented assessment of the consequences for sustainable development of the movement of people. The specter of mass migration is frequently used to mobilize action for environmental protection. Many climate change advocates raise the prospect of mass migration as a call for action. Some experimental research suggests that such prospects of undesirable migration flows incentivize mitigation actions (87, 88). By contrast, evidence from attitude surveys shows that host country populations tend to be more accepting of displaced people if they perceive them to be victims of climate change or other environmental disasters. This has been found from New Zealand accepting Pacific island migrants, and surveys of urban residents in Asian and African cities (89, 90). Beyond specific policies, the dominant discourses around migration in many ways constrain actions on sustainability.

Sustainability considerations need to be integrated into migration regulation and spatial planning in both origin and destination regions. For example, Fransen et al. (91) examine how the capacity of the 20 largest refugee settlements to support well-being of their inhabitants is affected by their exposure and resilience to extreme weather events. Their findings indicate that the relative high exposure of refugee settlements to extreme weather events challenges the viability of proposed refugee solutions and the localities of refugee settlements. Hauer et al. (73) demonstrate that with the demographic reality of more mobile populations able and willing to move as a result of climate risks, remnant populations in climate-exposed regions will also be aging populations, creating greater challenges for sustainable development of those places. In those circumstances, the demographic rebalancing of populations through inward migration has significant potential to support the pursuit of sustainability.

A further frontier for research is on how to ensure and facilitate migration as adaptation that not only alleviates immediate stresses but also enhances long-term prospects for sustainable development. Focusing on the processes of decision-making, Jarillo and Barnett (92) highlight the extent to which out-migration in three atoll islands in the Pacific represents climate adaptation. They find that Pacific islands are experiencing outward migration. But it is economic opportunities of migrants in their destination areas and the regulations to enable connections between origin and destination areas, that play a key role in stabilizing populations, facilitating belonging in those that remain and ultimately empowering climate change adaptation processes in those places. Hence, a practical focus on visa policies, return migration regulation, and strong diaspora links are in effect adaptation measures with the potential to support long-term sustainable development.

Sustainability science can fruitfully incorporate the migration dynamics into models of nature–society interactions and their implications for sustainable development. The challenge is substantial, given that the dominant narrative in the SDGs, for example, is that migrants are a “distinct social group at risk of being excluded from development” (p. 10) (76), rather than a potential transformative force in sustainable development pathways. Moreover, the sedentary discourse that underlies many of the SDG targets and indicators does not do justice to the reality of migration pathways and its role in transformations toward sustainability. Making migration visible can come about through explicit analysis of how migration drives demographic change within global scenarios, based on major regional flows (81, 93). But more fundamentally, too many framings of sustainability tend to conceive of migration as an anomaly from the norm, and a threat to stability and security (94, 95). In reality, involuntary movement of people under global change is both expected and inevitable given projections of future risks, while voluntary movement is desirable if the benefits of innovation, adaptation and collective well-being can be realized. A comprehensive research agenda that acknowledges and identifies how and under what conditions migration can contribute to sustainable development is needed to systematically incorporate scientific knowledge on migration and mobility in institutional and governance initiatives to promote sustainable and inclusive development.

## Data Availability

No original data or software were mobilised in support of this work. All public domain sources are referred to in the text.
